# Kinetics of the early events of GPCR signalling

**DOI:** 10.1016/j.febslet.2014.10.043

**Published:** 2014-12-20

**Authors:** Roslin J. Adamson, Anthony Watts

**Affiliations:** Biomembrane Structure Unit, Biochemistry Department, University of Oxford, South Parks Road, Oxford OX1 3QU, UK

**Keywords:** AC, adenylate cyclase, CHAPS, 3-[(3-cholamidopropyl)dimethylammonio]-1-propanesulfonate, CHS, cholesteryl hemisuccinate, DDM, dodecyl-β-d-maltoside, EDTA, ethylenediamine tetraacetic acid, FLAG-NTS1, FLAG-tagged NTS1, GDP, guanosine diphosphate, GTP, guanosine triphosphate, GPCR, G protein-coupled receptor, IPTG, isopropyl β-d-1-thiogalactopyranoside, NTS1, neurotensin receptor type 1, PI_3_K, phosphoinositide 3-kinase, PLC, phospholipase C, POPC, 1-palmitoyl-2-oleoyl-*sn*-glycero-3-phosphocholine, POPE, 1-palmitoyl-2-oleoyl-*sn*-glycero-3-phosphoethanolamine, POPG, 1-palmitoyl-2-oleoyl-*sn*-glycero-3-phospho-(1′-*rac*-glycerol), RU, response units, SCK, single cycle kinetics, G protein-coupled receptor, Electron microscopy, Surface plasmon resonance, Nanodisc, G protein

## Abstract

Neurotensin receptor type 1 (NTS1) is a G protein-coupled receptor (GPCR) that affects cellular responses by initiating a cascade of interactions through G proteins. The kinetic details for these interactions are not well-known. Here, NTS1-nanodisc-Gα_s_ and Gα_i1_ interactions were studied. The binding affinities of Gα_i1_ and Gα_s_ to NTS1 were directly measured by surface plasmon resonance (SPR) and determined to be 15 ± 6 nM and 31 ± 18 nM, respectively. This SPR configuration permits the kinetics of early events in signalling pathways to be explored and can be used to initiate descriptions of the GPCR interactome.

## Highlights

• Little is known of the kinetics of interactions between GPCRs and their signalling partners.
• NTS1 binds Gα_i1_ and Gα_s_ with affinities of 15 ± 6 nM and 31 ± 18 nM (SE), respectively.
• This SPR assay may be applicable to multiple partners in the signalling cascade.
• We provide the first direct evidence for GPCR-G protein coupling in nanodiscs.

## 1 Introduction

G protein-coupled receptors (GPCRs) constitute a large and diverse family of seven transmembrane receptors. Around 800 of these, the class A GPCRs, mediate responses of the cell to external stimuli such as hormones, photons, small molecules and peptides, through interactions with heterotrimeric G proteins. Ligand binding initiates a cascade of cell signalling events, beginning with a conformational change in the receptor that activates heterotrimeric G proteins. After activation by the receptor, Gα exchanges guanosine diphosphate (GDP) for guanosine triphosphate (GTP) in its binding pocket. Gα and Gβγ dissociate and signal through binding partners and second messengers to effect cellular response through diverse effectors, including adenylyl cyclase (AC), GTPases, phospholipase C proteins (PLCs), phosphoinositide 3-kinase (PI_3_K) and Ca^2+^ channels. Gα hydrolyses GTP to GDP and reassociates into the inactive heterotrimer with Gβγ. The signalling potential of GPCRs is amplified by their ability to bind various Gαβγs, which additionally may be composed of various combinations of the 21 Gα, 6 β, or 12 γ subunits [1].

The GPCR neurotensin receptor type 1 (NTS1) binds neurotensin (NT), a 13 amino acid peptide (ELYENKPRRPYIL) that acts as a neurotransmitter in the brain and as a local hormone in peripheral organs, with high affinity (*K*_D_ ∼1 nM) [2], [3]. NTS1 signals primarily through G_q_, which binds intracellular loop 3 of NTS1, but also through the inhibitory G_i1_ and the stimulatory G_s_, which bind near the C-terminus of the receptor [4]. NT modulates varied physiological responses, including appetite, stress and anxiety, analgesia [5], thermoregulation [6], maternal care [7] and dopaminergic signalling [2]. It thus plays an important role in conditions such as Parkinson’s disease, eating disorders, psychosis, drug addiction, pain and has also been implicated in colon cancer.

The only high-resolution structural knowledge of GPCR-G protein interactions is from the β_2_ adrenergic receptor-G protein complex structure solved by Rasmussen et al. [8], and the crystal structure of agonist-bound NTS1 was solved only recently [9]. Standard functional assays involving downstream effectors or radioactive GTPγS G protein activation to describe GPCR-G protein interactions do not assay the protein-protein interfacial interactions directly. Quantitative kinetic data of GPCR-G protein interactions comes from studies of the interactions of δ and μ-opioid receptors with G proteins using plasmon waveguide resonance [10], [11], [12]. Here, receptors were embedded in black lipid membranes (BLM) in order to mimic closer the native environment of the receptors. Few studies of the interactions of NTS1 with G proteins have been reported (all assayed by activation of the G protein) [4], [13], [14], and little is known of any of the kinetics of the subsequent signalling events. An understanding of the kinetics of interaction of GPCR receptors with their cognate G proteins, preferably under conditions that mimic the native lipid environment of the receptors, is essential for drug development that targets signalling pathways. Structural information and the determination of the affinity of binding would narrow the field of potential drug targets.

We have used surface plasmon resonance (SPR) to investigate the interactions of the α subunits of G_i1_ and G_s_ with NTS1 reconstituted into 10-nm size lipid discs termed nanodiscs [15], [16]. This avoids common problems encountered when studying membrane proteins using this method, such as detergent and glycerol giving rise to artefactual signals, whilst additionally enabling the study of the receptor in specific lipid mixes. To our knowledge, this is the first time that the G protein binding kinetics of a peptide-binding GPCR in a lipid membrane environment have been determined using SPR, and the first time that a GPCR in nanodiscs has been used as the analyte in SPR studies.

## 2 Materials and methods

### 2.1 Materials

Dodecyl-β-d-maltoside (DDM), 3-[(3-cholamidopropyl)dimethylammonio]-1-propanesulfonate (CHAPS) were purchased from Melford Laboratories and cholesteryl hemisuccinate (CHS) from Sigma. Palmitoyl-oleoyl phosphatidylcholine (POPC) and palmitoyl-oleoyl phosphatidylglycerol (POPG) were from Avanti Polar Lipids. All other reagents were analytical grade.

### 2.2 Protein expression and purification

#### 2.2.1 NTS1B purification

The NTS1B fusion construct has been described previously [17], [18]. The construct was modified to contain a FLAG tag (DYKDDDDK). NTS1B was expressed and purified as described previously [19], but phospholipids were omitted from the buffers and 10% glycerol was used in the final elution from the affinity column. TEV cleavage and affinity purification of cleaved NTS1 were performed as described [19], [20]. Approximately 1.0 mg FLAG-NTS1 was obtained from 80 g cells.

#### 2.2.2 MSP1D1 purification

The Membrane Scaffold Protein 1D1 (MSP1D1) construct was obtained from AddGene (Addgene plasmid 20061) [16]. The protein was expressed and purified according to [21] with modifications. Briefly, the protein was expressed at 37 °C using BL21(DE3) *Escherichia coli* cells (Calbiochem) in 2 L flasks containing 500 ml TB medium inoculated with 5 ml starter culture prepared as described, until the OD_600_ reached 1.6. Expression was induced with 1 mM IPTG and the cells were harvested by centrifugation (8000×*g*; 15 min). MSP1D1 purification was performed as described, with the exception that a cocktail of 2 μg/ml pepstatin A, 2 μg/ml leupeptin and 3 μg/ml aprotinin were used instead of phenylmethylsulfonyl fluoride (PMSF).

#### 2.2.3 G protein purification

The constructs for the alpha subunits of G_s_ and G_i1_ were kindly donated by Renaud Wagner (University of Strasbourg, France). Gα subunits were expressed and purified according to [22], with minor modifications. The His-tagged proteins were expressed using *E.* *coli* BL21(DE3) cells (Calbiochem) and purified using metal affinity chromatography on a 5 ml HisTrap High Performance column (GE Healthcare). Gα_i1_ was eluted from the column using a linear gradient of imidazole from 10 mM to 150 mM imidazole over 15 ml. Gα_s_ was eluted from the column in 10 mM steps up to 150 mM over 160 ml.

#### 2.2.4 FLAG-NTS1 reconstitution into nanodiscs

The protocols for reconstitution of membrane proteins into nanodiscs were followed in initial reconstitution attempts [15], [16], [21], [23], [24], [25], [26], but optimal ratios of MSP:FLAG-NTS1 and MSP:lipid were empirically determined. MSP:FLAG-NTS1 mol ratios of both 80:1 or 50:1 yielded fractions of homogeneously-sized nanodiscs, confirmed with negative stain electron microscopy (EM). The lipid:MSP ratio for either a 1:1 mix of POPC:POPG or a 3:1:1 mix of POPC:POPE:POPG with 25 mol% cholesterol was 65:1 for empty discs and 60:1 for loaded discs. Final concentrations of all components were approximately 160 μM MSP1D1, 8 mM lipid, 3 μM FLAG-NTS1, 16 mM sodium cholate, 2.6% glycerol. For empty discs, the volume of the reaction mixture was brought up to the same volume as the FLAG-NTS1 sample with the same DDM-containing buffer as FLAG-NTS1.

A calibrated Superdex 200 10/300 GL size exclusion column (GE Healthcare) was equilibrated in 50 mM Tris–HCl, pH 7.4, 100 mM NaCl, 5 mM MgCl_2_. Homogeneously-sized nanodiscs were separated from larger vesicles and aggregates at a flow rate of 0.4 ml/min.

Receptor-containing nanodiscs were enriched through the use of an anti-FLAG antibody column according to the directions (anti-FLAG M2 agarose, Sigma–Aldrich). Receptor-containing nanodiscs were eluted using 100 μg/ml FLAG® peptide (F3290, Sigma–Aldrich), dialysed extensively against Nanodisc SPR buffer (50 mM Tris–HCl, pH 7.4, 100 mM NaCl, 5 mM MgCl_2_), and concentrated to ∼1 μM using 100 000 MWCO Vivaspin centrifugal concentrator tubes (Sartorius).

#### 2.2.5 Surface plasmon resonance

**Fig. 1:**
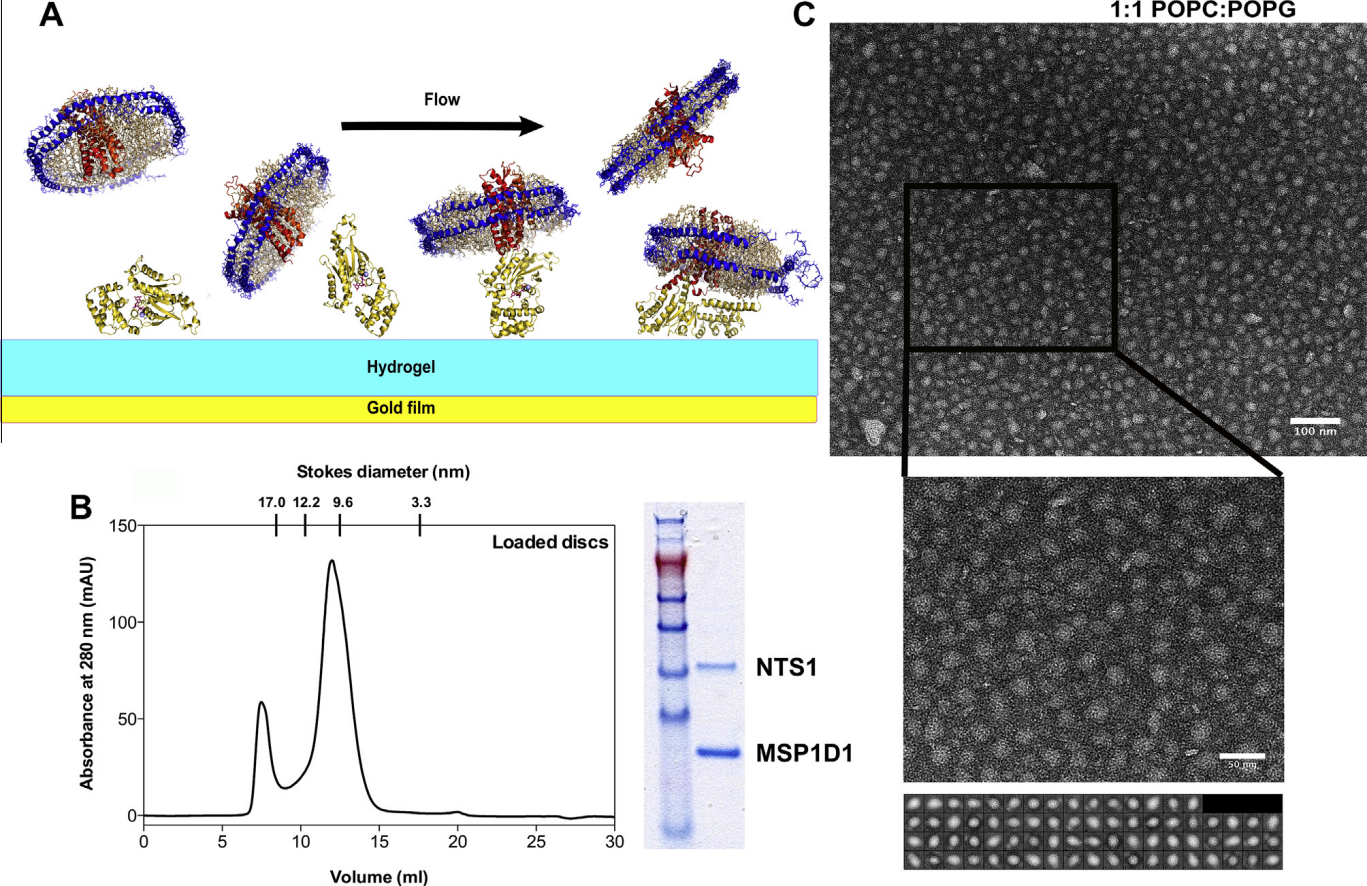
Nanodisc preparation and NTS1-G protein coupling determination. (A) Schematic representation of the experimental setup. G proteins (gold) were amine-coupled to a CM5 Biacore chip (GE Healthcare). FLAG-NTS1-loaded nanodiscs were injected over the sample and reference flow cells. The reference flow cell was activated and blocked or ovalbumin was amine-coupled to it. Empty discs were injected over the sample and reference flow cells as a reference. Single cycle kinetics using serial concentrations of FLAG-NTS1-nanodiscs was performed. Data were double-referenced. (B) Representative SEC profile for nanodisc purification. A peak composed of large aggregates and vesicles elutes in the void volume at approximately 7.5 ml, followed by the nanodisc peak at ∼12.5 ml, corresponding to a calibrated size of approximately 10 nm (left). SDS–PAGE of anti-FLAG enriched nanodiscs, showing approximately twice the amount of MSP1D1 compared to NTS1, which understains with Coomassie Brilliant Blue (right). (C) Negative stain EM images showing nanodisc sample. Nanodiscs prepared with a 1:1 ratio of POPC:POPG form homogeneous populations. Stain was 2% uranyl acetate. Reference-free class averages of 10–12 nm PC:PG discs (prepared using EMAN2 [54]) are shown. Box size is 18.5 nm. Scale bars are 100 nm (upper) and 50 nm (lower).

SPR was performed on a Biacore T100 instrument later upgraded to a T200 (GE Healthcare). Single cycle kinetics (SCK) were performed due to no suitable regeneration conditions being found for multiple cycles. Gα_s_ or Gα_i1_ were amine-coupled to the carboxymethylated surface of a CM5 chip (Biacore, GE Healthcare) using standard protocols. Briefly, G proteins were dialysed into 40 mM sodium phosphate buffer, pH 7.4 with 5 mM MgCl_2_. The calculated p*I* values for His-Gα_i1_ and His-Gα_s_ were 6.1 and 6.0 respectively (http://web.expasy.org/protparam). Coupling was most efficient at pH 5.0 for His-Gα_i1_ and pH 5.5 for His-Gα_s_, as determined by pH scouting on an unmodified chip surface. G proteins were diluted to 10 μg/ml in 10 mM sodium acetate pH 5.0 or 5.5. The chip was primed in HBS-N (10 mM HEPES, 150 mM NaCl, pH 7.4) and normalised with normalising solution (GE Healthcare). Coupling was as follows at 10 μl/min: 2 × 60 s injections of 50 mM NaOH, 420 s injection of NHS/EDC, 1000 s injection of Gα at appropriate pH, and finally the surface was blocked with a 420 s injection of ethanolamine. The reference flow cell was either simply activated and blocked with 2 × 60 s injections of 50 mM NaOH, 420 s injection of NHS/EDC, and 420 s injection of ethanolamine, or ovalbumin was amine-coupled to the surface as above, with 840 s injection of 10 μg/ml in 10 mM sodium acetate, pH 4.0. The chip was extensively washed at 50 μl/min for 1–2 h, then primed in Nanodisc SPR buffer. NT-bound (NT in excess (5 μM)), anti-FLAG-enriched NTS1-nanodiscs and empty nanodiscs were serially diluted five times from 660 nM or 400 nM. SCK programmes were performed at 30–50 μl/min, using the empty nanodiscs as a buffer reference. Three 60 s start-up injections of Nanodisc SPR buffer were followed by serial injections of nanodiscs for 90–120 s. Fig. 1A shows a schematic diagram of the experiment.

Data were confirmed by switching the roles of nanodiscs and Gα subunits to ligand (the surface-bound molecule in SPR terminology) and analyte (in solution) respectively.

SPR data was analysed using the BiaCore T100 or T200 BiaEvaluation software (Biacore). Data was double-referenced and 1:1 and heterogeneous ligand binding fits were applied.

## 3 Results

### 3.1 Nanodisc formation

Nanodisc formation efficiency depended on the lipid:MSP1D1:NTS1 ratio. Lower amounts of lipid were required when higher ratios of NTS1 incorporated into discs were needed. High ratios of MSP1D1:FLAG-NTS1 (50:1 mol:mol) were used to ensure insertion of primarily monomers into the nanodiscs. Assuming a Poisson distribution of FLAG-NTS1 into the discs [27], with this 50:1 ratio over 96% of discs would be empty, 3.8% would contain one receptor, and less than 0.08% would contain two receptors. After enrichment of loaded nanodiscs using the FLAG tag on NTS1 and an anti-FLAG column, 2% of discs would contain two receptors. In all cases though, some level of large aggregates was present in the nanodisc reaction mixture, which was separated from the homogeneous nanodisc population using SEC (Fig. 1B). The peak fractions corresponding to a disc size of ∼10 nm were pooled, anti-FLAG purified if required, concentrated and dialysed. As expected, the discs contained twice the amount of MSP1D1 as NTS1, and were pure on gels (Fig. 1B). EM confirmed size homogeneity of the populations (Fig. 1C). Specific activity of the NTS1-nanodiscs was determined at ∼5% by a radioligand binding assay.

Diameters for the FLAG-NTS1-loaded PC:PG-containing discs were 10 nm, and slightly smaller for the PPPC-containing discs at 9.5 nm. Empty discs tended to be smaller (∼0.6 nm) than the respective loaded discs. The diameters of nanodiscs calculated from the standards of a calibrated gel filtration column correspond to average molecular masses of approximately 200 ± 15 kDa for the loaded PC:PG discs, and for empty discs, 10–20 kDa less. Correspondingly, PPPC discs were 160–180 kDa, with empty discs up to 15 kDa lighter. The Stoke’s radius assumes a spherical particle, thus overestimating the mass of a disc-shaped object, and so the number of lipid molecules calculated from the area of the disc is likely to be lower than that calculated for the molecular mass. Taking the PC:PG discs as an example, and using lipid areas of 0.56 nm^2^ for POPG and 0.66 nm^2^ for POPC [28], subtracting 1 nm from the radius of the disc for the diameter of an α-helix (the MSP), and subtracting the area of NTS1 based on a radius of approximately 2 nm, it can be calculated that there are approximately 62 lipid molecules per leaflet (or 70 for empty discs). This is the number of lipid molecules put into the reaction mixture to form nanodiscs for most cases, where a 1:60 MSP:lipid mol ratio was used. This also correlates well with other data indicating a typical lipid number of 62 lipid molecules per leaflet for POPC only discs, where a slightly larger lipid area for POPC was used [15], [16]. NTS1 would therefore be surrounded by just over three complete annuli of lipid molecules in the nanodisc. Calculating the number of lipid molecules from the total molecular mass given by the Stoke’s radius (200 kDa) would give almost 20 lipid molecules (average MW of 750 Da) more per nanodisc.

### 3.2 FLAG-NTS1-nanodisc-G protein coupling

**Fig. 2:**
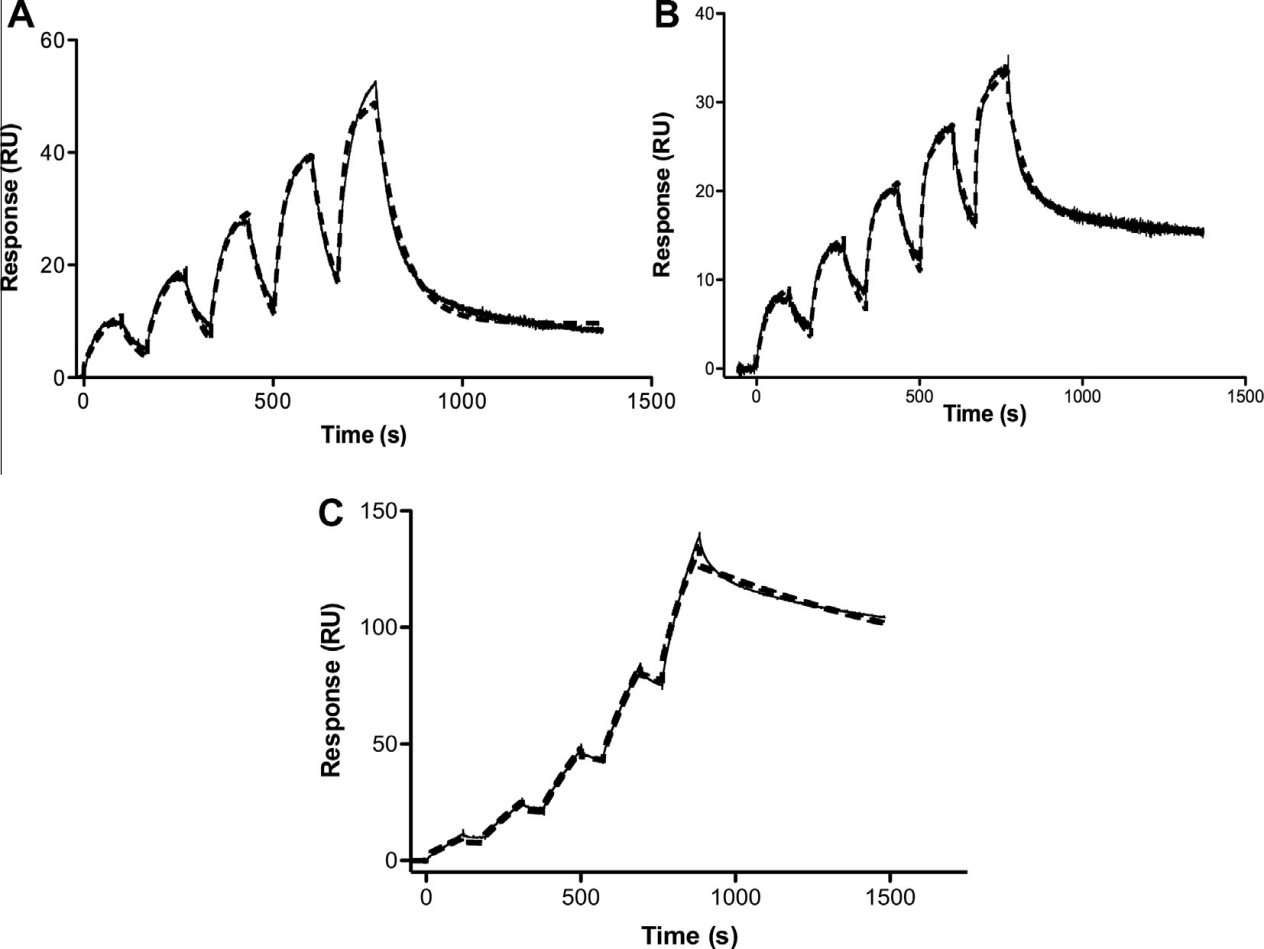
SPR traces of GPCR G coupling. (A) and (B) FLAG-NTS1-PC:PG nanodiscs coupling to His_6_-G_s_ or His_6_-G_i1_ immobilised on a CM5 chip. Approximately 7000 RU G_s_ was amine-coupled to a CM5 Biacore chip (GE Healthcare) in 10 mM sodium acetate, pH 5.5 (A). Approximately 13 000 RU G_i1_ was amine-coupled to a CM5 Biacore chip (GE Healthcare) in 10 mM sodium acetate, pH 5.0 (B). The reference flow cell was activated and blocked. Serial concentrations of 41.25–660 nM (A) and 25–400 nM (B) nanodisc-reconstituted and ligand-bound FLAG-NTS1 were injected over the chip surface. Empty nanodiscs at the same concentrations were injected as a reference, and data were double-referenced. The affinity of G_s_ (A) for FLAG-NTS1 in nanodiscs was 9 nM in this instance, and 9 nM for G_i1_ (B). (C) His_6_-G_s_ coupling to FLAG-NTS1-PC:PG nanodiscs captured on an L1 chip. FLAG-NTS1-PC:PG nanodiscs (2500 RU) and empty PC:PG nanodiscs (2000 RU) were captured in FC 4 and 3, respectively, of an L1 chip by an 800-s injection at 5 μl/min. The chip was thoroughly washed in running buffer at 50 μl/min for 30–60 min. Serial dilutions of 1000 nM (62.5, 125, 250, 500, 1000 nM) His_6_-G_s_ were injected across the flow cells for 150 s per concentration at 50 μl/min. The data (solid lines) were fitted with a 1:1 Langmuir binding model as well as a heterogeneous ligand binding model (dashed lines), giving *K*_D_ values of 65 nM for the 1:1 fit and 0.5 and 80 nM for *K*_D1_ and *K*_D2_ respectively. The *χ*^2^ values for the fits were 4.4 and 3.3, respectively.

G proteins were amine-coupled to the SPR chip or nanodiscs were captured on an L1 chip (Fig. 2A–C). Between 5000 and 13 000 RU were coupled and the surface rigorously washed to minimise baseline drift. Injections of nanodiscs over G protein showed some concentration-dependence in the signal at higher concentrations, which was partially abolished by lowering the top concentration of analyte (Fig. 2A and B). Single cycle kinetics (SCK) programmes were used. Empty nanodiscs were used as a reference in order to match the refractive index of the sample solution, since it contained both protein and lipid. To fit the data, a standard 1:1 Langmuir binding model was used initially, but ultimately, a heterogeneous ligand binding (HLB) model was found to be more appropriate, given the non-specific nature of amine coupling (inbuilt BiaEvaluation software, Biacore). HLB global fits to the single cycle kinetics data produced the kinetic parameters listed in Table 1.

**Table 1:** Averaged kinetic parameters for Gα coupling to FLAG-NTS1-PC:PG nanodiscs.

	His_6_-Gα_s_			His_6_-Gα_s_ with GTPγS			His_6_-Gα_i1_		
	Mean	SEM^a^	N^b^	Mean	SEM	N^b^	Mean	SEM	N^b^
*k*_a1_ (M^−1^ s^−1^)	1.9 × 10^5^	1.9 × 10^3^	12	1.4 × 10^5^	680	4	3.2 × 10^5^	340	6
*k*_d1_ (s^−1^)	2.4 × 10^−3^	4.2 × 10^−5^	12	1.7 × 10^−3^	5.4 × 10^−5^	4	1.1 × 10^−2^	8.4 × 10^−6^	6
*K*_D1_ (nM)	31	18	12	72	23	4	15	6	6
*k*_a2_ (M^−1^ s^−1^)	4.6 × 10^5^	3.0 × 10^4^	10	8.9 × 10^4^	1.8 × 10^3^	4	1.4 × 10^5^	2.8 × 10^3^	6
*k*_d2_ (s^−1^)	4.4 × 10^−1^	7.5 × 10^−3^	10	7.3 × 10^−2^	5.1 × 10^−4^	4	1.6 × 10^−2^	1.1 × 10^−4^	6
*K*_D2_ (nM)	470	130	10	880	160	4	330	170	6
*R*_max1_	29	16	12	30	22	4	29	15	6
*R*_max2_	33	10	10	21	4.6	4	37	15	6

A one-tailed *t*-test comparing His_6_-Gα_s_ with GTPγS and His_6_-Gαi1 with His_6_-Gα_s_ in pairs established that there were no significant differences between either of the two paired datasets (*p* > 0.05).

a Standard error of the mean.

b Number of experiments.

## 4 Discussion

### 4.1 GPCR-G protein signalling

For the first time, the direct interactions of a GPCR in nanodiscs with G proteins have been investigated using SPR. The kinetics of this interaction and the affinity of binding are of much interest. These are aspects of G protein coupling, or any downstream events, that have rarely been assayed directly. Typical assays for G protein-GPCR coupling follow the activation of the G protein through radioactive assays using [^35^S]GTPγS, or by assaying cAMP or Ca^2+^ levels. Knowing the affinity of a G protein for a GPCR, and the differential affinities of the various G proteins for the same GPCR, and then isolating the residues involved in the interaction and potentially studying how different βγ subunits influenced the interaction, would be of immense use for the development of drugs targeting specific signalling pathways or protein-protein interfaces (druggable interfaces). An additional level of complexity that needs to be unravelled would be how different agonists affect the affinities and rates of binding of G proteins to GPCRs, and whether the type of lipid environment of the receptor has any influence on these parameters.

### 4.2 Novel use of nanodiscs to detect signalling

Reconstituting NTS1 into nanodiscs eliminated bulk signals caused by detergent and/or glycerol, which are required for maintenance of receptor function and stability when extracted from membranes. Drift from the chip was also eliminated, because the receptor itself could be used as the analyte rather than tagged to NT as the ligand. Further advantages of this configuration were that NTS1 was in a bilayer, the nanodiscs could be dialysed and concentrated, and the NTS1-nanodiscs could also be used as the ligand, by capturing them on an L1 chip. Using the empty nanodiscs as the reference “blank” ensured that the closest fitting blank possible was being used to subtract any non-specific binding signal.

### 4.3 Scope

GPCR structure and function are ideally assayed in a membrane environment [29], [30], [31]. However, lipid membranes and membrane-mimetic environments do not readily lend themselves to most biophysical methods. SPR is by now a well-established real-time, label-free means of robustly determining the binding constants and affinities of proteins for antibodies, ligands or other binding partners, and the binding of NTS1 to NT in detergent has already been demonstrated [3], [32]. A number of other studies have investigated GPCR-ligand or drug interactions, reconstitution of GPCRs on SPR chips, or GPCR-G protein interactions in detergent [33], [34], [35], [36], [37], [38], [39], [40], [41].

Nanodiscs have been used as the analyte in SPR studies previously. Some instances were found [27], [42], [43], but to date no other SPR study has used nanodiscs to investigate GPCR-G protein coupling. The affinity constants of NTS1 in nanodiscs determined for both Gα_i1_ and Gα_s_ were in the low nanomolar range, implying high affinity for the receptor. There was statistically no difference in the affinities, or in the affinity of GTPγS-bound Gα_s_ for the receptor. Reducing heterogeneity in the system may prove this not to be the case, but stable receptor-G protein-GTPγS complexes have been observed [44].

Our experiments were performed in the presence of ligand. However, Alves et al. used plasmon waveguide resonance, a variant of SPR, to study the affinities of various Gα_i_ and Gα_o_ proteins for the δ-opioid receptor in the presence and absence of ligand. The affinities were found to be ligand- and βγ-subunit-dependent [12], [45]. Thus, within the setting of the cell there is enormous potential for broad scope of receptor function, depending on multiple parameters. With every additional parameter, sensitivity and subtlety of function grows. GPCRs are able to bind many different ligands and G proteins. If the affinity of each different G protein for the receptor is modulated by the type and presence or absence of ligand, the type and presence or absence of βγ subunit; and the affinity of GTP(γS), which activates the G protein, for the G protein alters according to the above parameters, the scope for function is significant. Add to this the potential for homo- and hetero-dimerisation of the receptor and it becomes increasingly clear why GPCRs are responsible for, and capable of controlling, so many of the essential and critical cell functions, and why any defect of function anywhere along the signalling pathway can have such a profound influence on the health of the organism.

The ability to explore, relatively rapidly, the affinities of various G proteins for their cognate receptors is important for many reasons, including testing the effects of mutations to conserved residues within the C-terminal α-helix of the G protein, or within the residues of a GPCR that are expected to bind the G protein, or the effects of different lipid environments on the coupling affinity and rate of binding of a GPCR to G proteins, or the effect of the βγ-subunit on the coupling, is immensely useful for later clinical research for drug-targeting of signalling pathways. These are aspects currently under study.

**Fig. 3:**
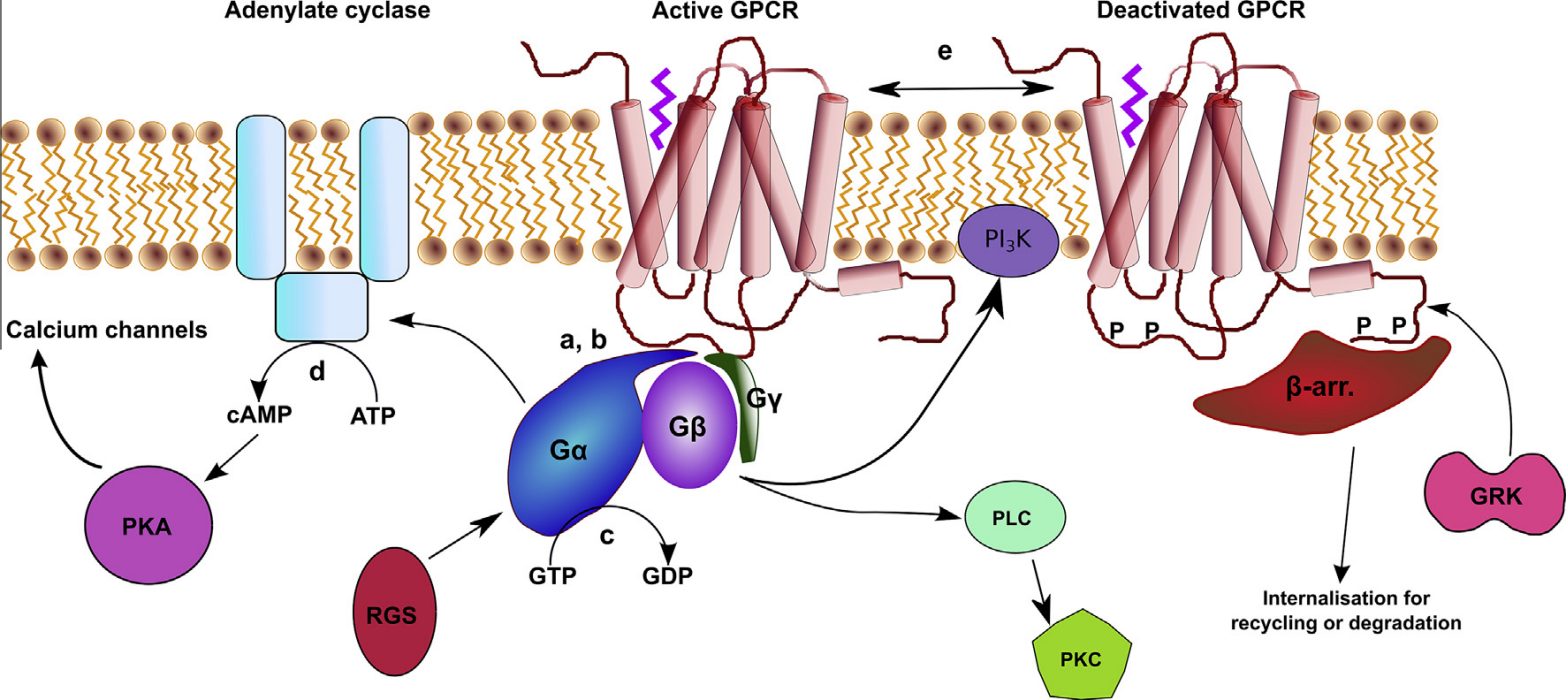
Kinetics of the GPCR interactome. Some of the initial steps in the GPCR signalling pathway. The GPCR activates a heterotrimeric G protein (a, b) via the GTPase domain of the Gα subunit, which is regulated by regulators of G protein signalling (RGS), causing hydrolysis of GTP to GDP (c). The heterotrimer dissociates into α and βγ subunits. The hydrolysis of ATP to cyclic AMP (cAMP) (d) is catalysed by interactions of Gα with adenylate cyclase, which regulates Ca^2+^ channels via activation of protein kinase A (PKA) by cAMP. The Gβγ subunits activate phosphatidylinositide 3-kinase and phospholipase C (PLC). PLC cleaves phosphatidylinositol 4,5-bisphosphate (PIP_2_) into inositol 1,4,5-trisphosphate (IP_3_) and diacyl glycerol (DAG) which activate the release of Ca^2+^ from the endoplasmic reticulum and the activation of protein kinase C (PKC). PKA and G protein-coupled receptor kinases (GRKs) phosphorylate the GPCR, leading to coupling of the receptor to arrestin and subsequent down-regulation of the receptor by internalisation for recycling or degradation in lysosomes. (a, b) Gα_i1_ and Gα_s_ affinities for NTS1 of 15 ± 6 nM and 31 ± 18 nM (SE), respectively (this study). (c) BODIPY-GTP hydrolysis *K_m_* value was 120 ± 60 nM [55]; GTPγS binding *k*_app_ = 0.027 min^−1^[56]. (d) Basal activity ∼20–65 pmol cAMP/min/mg [57], *k*_obs_ of ∼1 × 10^−3^ − 6 × 10^−4^ s^−1^[58]. (e) Spontaneous diffusion-interaction on the ms timescale [59], with dissociation rates of 1.3 s^−1^[60], and a *K*_D_ of 2–20 nM [61].

Mapping the GPCR interactome is going to be challenging due to its inherent complexity [46], [47], [48] (Fig. 3), and here we have shown one approach to understanding the biology associated with cellular responses controlled through GPCRs. This importance has not escaped the pharmaceutical industry, as demonstrated through its continuance to focus on GPCRs as drug targets [49], [50], [51], [52], [53].
