# Supplementary material for: Kinetics of the early events of GPCR signalling
Source: FEBS Lett. 2014 Dec 20;588(24):4701–7. doi: 10.1016/j.febslet.2014.10.043 (PMC4266533; doi:10.1016/j.febslet.2014.10.043)
Supplement: Supplementary information [file mmc1.docx]

**Kinetics of the early events of GPCR signalling**

Roslin J Adamson^1^ and Anthony Watts^1^*

^1^ Biomembrane Structure Unit, Biochemistry Department, University of Oxford, South Parks Road, Oxford, OX1 3QU, UK

To whom correspondence should be addressed: Anthony Watts, Biomembrane Structure Unit, Biochemistry Department, University of Oxford, South Parks Road, Oxford, OX1 3QU, UK. Tel: (+44)(0)1865-613-248, [anthony.watts@bioch.ox.ac.uk](mailto:anthony.watts@bioch.ox.ac.uk)

**Supplementary information**

**Materials and Methods**

TEV NIa protease purification

The hexahistidine-tagged tobacco etch virus NIa protease (TEV protease) construct was kindly donated by Dr Huanting Liu and Dr Jim Naismith (University of St. Andrews). *Escherichia coli* BL21(DE3) cells (Calbiochem) harbouring the pETNIP plasmid containing the His_6_-TEV NIa protease sequence were induced with 0.4 mM isopropyl β-D-1-thiogalactopyranoside (IPTG) and grown for 20 hours in 2xYT medium containing 50 μg/ml of kanamycin (20 °C; shaking speed 120 rpm). The cells were lysed by sonication and cell debris was removed by two centrifugations (20 000 g, 20 min and 70 000 g, 30 min). TEV protease was purified as described [[1](#_ENREF_1)], but 50 mM Tris buffer, pH 7.4 was used instead of phosphate buffer.

Gα subunit activity

The activity of the Gα subunits was checked using fluorescence polarisation on a Pherastar Plate Reader (BMG). Aliquots of BODIPY-GTPγS were injected into wells containing Gα_s_ or Gα_i1_ such that the final concentrations of fluorophore and G protein were 25 nM and 250 nM respectively. The polarisation signal was monitored over a time course of 30 minutes.

FLAG-NTS1 reconstitution into nanodiscs

Two lipid mixtures, one consisting of a 3:1:1 mol ratio of POPC:POPG:POPE with 25 mol % cholesterol, as well as a 1:1 mol ratio of POPC:POPG were used. A total phosphorus assay was used to determine concentrations of lipid stocks in chloroform (Chen et al., 1956). Lipid films of 25-50 μmol lipid were dried under a nitrogen stream and residual solvent removed under vacuum overnight. Lipid stocks were made up at 40-60 mM in 100 mM sodium cholate prepared in 50 mM Tris-HCl, pH 7.4, 100 mM NaCl, 1 mM EDTA. The solution was freeze-thawed three times and briefly (~1 min) immersed in a sonicating water bath. An MSP:lipid mol ratio of 1:60 or 1:55 was used for FLAG-NTS1-loaded discs, and 1:65 for empty discs. Reaction mixtures contained 9.6-27.6 μmol lipid, 0.16-0.46 μmol MSP1D1, and 2.0 to 9.2 nmol FLAG-NTS1, depending on the size of the preparation and ratios chosen. A typical reaction contained 27.6 μmol sodium-cholate-solubilised lipid, 9.2 nmol FLAG-NTS1, and 0.46 μmol MSP1D1 added in that order, in a final volume of approximately 3 ml.

After addition of all components, the nanodisc reaction was rotated at 4 rpm at 4 °C for 1 h. Thereafter, 1 g per ml Biobeads® SM (Bio-Rad) was added and the reconstitution allowed to proceed overnight (16 hours). The reaction volume was placed into 0.3 g/ml fresh Biobeads® SM for an additional 2 hours. Thereafter the sample was concentrated to 500 μl using 100 000 MWCO Vivaspin 6 centrifugal concentrator devices (Sartorius). Centrifugation for 10 minutes at 10 000 rpm, 4 °C, in a benchtop Beckman Avanti™ 30 centrifuge followed by centrifugal filtration through 0.22 μm membranes (Nanosep) removed large aggregates prior to size exclusion chromatography.

Electron microscopy

Negative stain transmission electron microscopy (TEM) was used to confirm formation of nanodiscs and population size homogeneity. Formvar-carbon coated 300 mesh copper grids were glow-discharged for 30 seconds. Purified nanodiscs (4 μl) were adsorbed to the grids for 60 seconds, blotted briefly and then stained for 10 seconds with 2 % uranyl acetate. Grids were viewed at 59 000 x magnification on an FEI Tecnai T12 elelctron microscope at an accelerating voltage of 80 keV and imaged on a Gatan Eagle 4k by 4 k CCD detector.

**Results**

*Kinetic information for Figure 2A and B.*

The association rate, *k*_a1,_ of FLAG-NTS1 nanodiscs binding to Gα_s_ was 1.6 x 10^4^ ± 1.5 x 10^2^ M^-1^s^-1^, and the dissociation rate, *k*_d1_, was 1.4 x 10^-4^ ± 6.1 x 10^-6^ s^-1^. *k*_a2_ was 4.1 x 10^5^ ± 7.4 x 10^3^ M^-1^s^-1^. *k*_d2_ was 1.6 x 10^-2^ ± 2.5 x 10^-4^ s^-1^, and *K*_D2_ = 39 nM. For Gα_i1_ (B), *k*_a,_ = 7.7 x 10^2^ ± 34 M^-1^s^-1^, and *k*_d_, = 7.2 x 10^-6^ ± 4.3 x 10^-7^ s^-1^. *k*_a2_ = 9.6 x 10^4^ ± 5.3 x 10^2^ M^-1^s^-1^, *k*_d2_ = 1.4 x 10^-2^ ± 4.3 x 10^-5^ s^-1^, with *K*_D2_ = 150 nM.

*SPR data fitting*

Most of the data obtained for the NTS1-nanodisc-G protein-coupling experiments could be fitted relatively well with a heterogeneous ligand binding model. The χ^2^ values were low. The standard errors and statistical tests for parameter significance were within the acceptable ranges, and the residuals for the fits were also within acceptable ranges for a good fit. The kinetic data was reproducible between experiments, within error, and changing the roles of the binding partners did not alter the outcome. The fraction of data with the best fits, representing possibly one third to one half of the total data is presented here (Table 1, main text). The heterogeneous ligand binding model assumes two equivalent and independent sites on the ligand, and is frequently used when the method of immobilisation is amine coupling, due to its non-specific nature. The ligand may be immobilised in many different orientations depending on the number and location of Lys residues, and binding sites may be occluded. An examination of the Lys residues in Gα_i1_ and Gα_s_ shows some differences between the two, potentially accounting for some of the differences seen in the immobilisation efficiency of the proteins and the variations in signal obtained for the two. Gα_s_ has a patch in the GTPase domain that is clear of Lys residues, unlike the same area in Gα_i1_, and this is where the C-terminal α-helix that binds the receptor is located. Chances that receptor binding would be occluded by this portion of the molecule being covalently coupled to the chip are smaller than those for Gα_i1_, which has Lys residues clustered in both domains. Gα_s_ has 5 fewer Lys residues (25) than Gα_i1_ (30). However, within the system studied, ligand immobilisation is not the only potential source of heterogeneity. Other possible sources are: not all of the receptor may be ligand-bound; not all of the Gα may be GDP-bound, since the Gα storage buffer was not supplemented with GDP; and some of the nanodiscs could contain NTS1 dimers. GPCRs and G proteins are known to be able to form a pre-coupled complex prior to ligand-binding, which increases affinity for the ligand [[2](#_ENREF_2),[3](#_ENREF_3)]. Is it possible that non-ligand-bound NTS1 pre-coupled to Gα on the chip surface with a lower binding affinity? If so, this would affect the data fit. There is a small chance that some of the discs contain dimers. Reconstitution of target protein into nanodiscs follows a Poisson distribution [[4](#_ENREF_4)], such that at the 1:50 MSP:NTS1 ratio used for reconstitution here, 96 % of nanodiscs would be empty, close to 3.8 % would contain one receptor and a tiny fraction would contain two receptors. After enrichment, this fraction would still constitute a negligible proportion, although the tendency for NTS1 to form dimers or not may skew this distribution. This could give differential binding parameters.

*NTS1-nanodisc-NT binding.*

To test the activity of the NTS1-nanodiscs, CysNT was thiol-coupled to a CM5 chip and nanodiscs injected across the flow-cells (Figure 1). The binding response was low, given that not all of the protein is active, but the binding affinity obtained was in keeping with published results.

**Figure 1.** **NTS1-nanodisc-NT binding kinetics.**

Single cycle kinetics sensorgram of FLAG-NTS1-loaded nanodiscs binding to CysNT thiol-immobilised on a CM5 chip. The *K*_D_ is similar to those determined in the literature, but much higher concentrations of nanodiscs were needed than for NT coupling in detergent, and the response is very weak. This may imply a steric hindrance to binding in nanodiscs on the chip, and that NTS1 is not very active in PC:PG (~5% active). The results from one experiment are shown above. A 1:1 Langmuir model was used. The χ^2^ value was 0.1. The mean from two experiments was 1.3 ± 0.6 nM (SE).

[1] Liu, H. and Naismith, J.H. (2009). A simple and efficient expression and purification system using two newly constructed vectors. Protein Expr Purif 63, 102-11.

[2] Nobles, M., Benians, A. and Tinker, A. (2005). Heterotrimeric G proteins precouple with G protein-coupled receptors in living cells. Proc Natl Acad Sci U S A 102, 18706-11.

[3] Gales, C., Van Durm, J.J., Schaak, S., Pontier, S., Percherancier, Y., Audet, M., Paris, H. and Bouvier, M. (2006). Probing the activation-promoted structural rearrangements in preassembled receptor-G protein complexes. Nat Struct Mol Biol 13, 778-86.

[4] Ritchie, T.K., Grinkova, Y.V., Bayburt, T.H., Denisov, I.G., Zolnerciks, J.K., Atkins, W.M. and Sligar, S.G. (2009). Chapter 11 - Reconstitution of membrane proteins in phospholipid bilayer nanodiscs. Methods Enzymol 464, 211-31.
